# A Three-Dimensional Xeno-Free Culture Condition for Wharton’s Jelly-Mesenchymal Stem Cells: The Pros and Cons

**DOI:** 10.3390/ijms24043745

**Published:** 2023-02-13

**Authors:** Benson Koh, Nadiah Sulaiman, Mh Busra Fauzi, Jia Xian Law, Min Hwei Ng, Too Lih Yuan, Abdul Ghani Nur Azurah, Mohd Heikal Mohd Yunus, Ruszymah Bt Hj Idrus, Muhammad Dain Yazid

**Affiliations:** 1 Centre for Tissue Engineering & Regenerative Medicine, Faculty of Medicine, Jalan Yaacob Latif, Cheras, Kuala Lumpur 56000, Malaysia; 2 Universiti Kebangsaan Malaysia Medical Centre, Jalan Yaacob Latif, Cheras, Kuala Lumpur 56000, Malaysia; 3 Ming Medical Sdn Bhd, D3-3 (2nd Floor), Block D3 Dana 1 Commercial Centre, Jalan PJU 1a/46, Petaling Jaya 47301, Malaysia; 4 Department of Obstetrics and Gynaecology, Faculty of Medicine, Universiti Kebangsaan Malaysia Medical Centre, Jalan Yaacob Latif, Cheras, Kuala Lumpur 56000, Malaysia; 5 Department of Physiology, Faculty of Medicine, Universiti Kebangsaan Malaysia Medical Centre, Jalan Yaacob Latif, Cheras, Kuala Lumpur 56000, Malaysia

**Keywords:** Wharton’s jelly mesenchymal stem cells, human platelet lysate, xeno-free culture, microcarriers, three-dimensional culture

## Abstract

Xeno-free three-dimensional cultures are gaining attention for mesenchymal stem cell (MSCs) expansion in clinical applications. We investigated the potential of xeno-free serum alternatives, human serum and human platelet lysate, to replace the current conventional use of foetal bovine serum for subsequent MSCs microcarrier cultures. In this study, Wharton’s Jelly MSCs were cultured in nine different media combinations to identify the best xeno-free culture media for MSCs culture. Cell proliferation and viability were identified, and the cultured MSCs were characterised in accordance with the minimal criteria for defining multipotent mesenchymal stromal cells by the International Society for Cellular Therapy (ISCT). The selected culture media was then used in the microcarrier culture of MSCs to determine the potential of a three-dimensional culture system in the expansion of MSCs for future clinical applications, and to identify the immunomodulatory potential of cultured MSCs. Low Glucose DMEM (LG) + Human Platelet (HPL) lysate media appeared to be good candidates for replacing conventional MSCs culture media in our monolayer culture system. MSCs cultured in LG-HPL achieved high cell yield, with characteristics that remained as described by ISCT, although the overall mitochondrial activity of the cells was lower than the control and the subsequent effects remained unknown. MSC microcarrier culture, on the other hand, showed comparable cell characteristics with monolayer culture, yet had stagnated cell proliferation, which is potentially due to the inactivation of FAK. Nonetheless, both the MSCs monolayer culture and the microcarrier culture showed high suppressive activity on TNF-α, and only the MSC microcarrier culture has a better suppression of IL-1 secretion. In conclusion, LG-HPL was identified as a good xeno-free media for WJMSCs culture, and although further mechanistic research is needed, the results show that the xeno-free three-dimensional culture maintained MSC characteristics and improved immunomodulatory activities, suggesting the potential of translating the monolayer culture into this culture system in MSC expansion for future clinical application.

## 1. Introduction

MSCs have been studied extensively for their regenerative properties. Unlike embryonic pluripotent stem cells, MSCs have emerged as the preferable choice of stem cells in regenerative medicine, most probably due to the lack of ethical, histocompatibility, and teratoma-formation issues, and their ability to differentiate into multi-lineages such as adipocytes, osteocytes, chondrocytes, and myocytes [1,2,3]. MSCs are also easily available from various adult tissue sources, such as blood or adipose tissue, the dermis, muscle, dental pulp, and Wharton’s jelly [4,5,6].

The demand for stem cells in clinical studies is growing rapidly, especially during the transition from cell-based therapy to the development of cell-free therapy. Aside from their multilineage potency, the immunomodulatory and anti-inflammatory properties of MSCs have been extensively studied not only for regenerative medicine, but also for the emerging COVID-19 [7,8,9]. The current pandemic is continuing to challenge medical health systems all over the world, and the future outlook remains discouraging. The use of MSCs and their extracellular vesicles gained attention from clinicians during the COVID-19 pandemic due to their strong immunomodulatory properties, as mentioned. There are increasing numbers of clinical trials using MSCs, registered in clinicaltrials.gov, in search of a way to relieve COVID-19 patients from complications, especially the aggressive inflammatory response which leads to lung injury, multi-organ failure, and the unfavourable prognosis of severe COVID-19 [9,10,11,12,13,14]. Despite the increase in MSCs applications, the quality and the safety of MSCs still remain debatable due to the inconsistency in upscale culture conditions.

In recent decades, innovations in cell culture-related consumables aimed to improve and provide alternatives to the existing monolayer culture flasks. Three-dimensional culture systems and microcarriers were introduced to address current problems in MSC expansion due to surface area restriction. Microcarriers are small, sphere-like structure beads that provide surface matrices that enable the attachment of adherent cells to form cell-microcarrier complexes suspended in growth medium [15]. This culture system is relatively flexible, as it provides large culture surfaces for higher cell yield and is able to integrate into existing three-dimensional bioprocess manufacturing systems. Multiple studies have demonstrated successful MSC up-scale expansion using microcarriers in three-dimensional culture systems.

Foetal bovine serum (FBS) and Dulbecco’s Modified Eagle’s Medium (DMEM) is a common media combination used in MSC culture. As with other xenogeneic products, the use of FBS is undesirable due to the potential transmission of infectious diseases and immunorejection reactions after clinical transplantation due to the xenogeneic proteins transmitted from FBS to MSCs during the culture process [16,17,18,19]. In fact, as much as 20 to 50% of commercial FBS is virus-positive [20], which could be a potential biohazard risk in clinical studies. A single preparation of 108 MSCs grown in FBS may also carry approximately 7 to 30 mg of xenogeneic proteins [21], which will greatly affect the purity of the product if extracellular vesicles are taken into account. This is not to mention the batch-to-batch variation that is frequently observed in FBS, potentially adding a factor that causes inconsistency in the cellular therapy product. The processing procedure in FBS production contributes as the key factor in controlling batch variation, while the poor regulation of FBS production may lead to cases of non-conformity [22], greatly affecting the results in cell cultures. Studies observed that the content of endotoxin and growth hormone varied widely in batches of FBS, resulting in the inconsistency in cell expansion [23,24]. Because of this, the search for an ideal FBS alternative is an important step to consider in MSCs expansion.

The use of xeno-free culture media in in vitro manipulation of MSCs for clinical applications has therefore become a crucial step in order to yield high quality MSC cultures, but without an increase in hazardous risks. Human-based serum supplements, typically human serum (HS) and human platelet lysate (HPL). are currently being explored as an alternative to FBS in conventional culture systems [25,26,27,28,29]. In this study, we examined the possibility of HPL and HS as an alternative to FBS-supplemented medium, together with other basal media, to determine a suitable culture media for Wharton’s jelly-derived MSC (WJMSC) microcarrier culture. In addition to cell expansion, the immunomodulatory ability of xeno-free media-cultured MSCs will also be evaluated in this study.

## 2. Results

### 2.1. Human Platelet Lysate as a Reliable FBS Substitute for Xeno-Free Culture

#### 2.1.1. Human Platelet Lysate Culture Media Alters WJMSCs Morphology and Growth in the 2D Culture Setting

**Figure 1:**
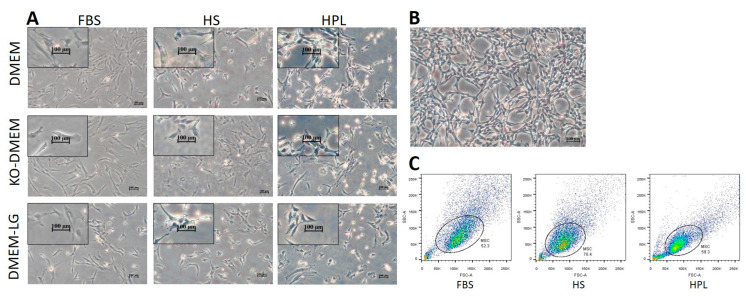
Morphological characteristics of Wharton’s jelly mesenchymal stem cells (WJMSCs). (**A**) WJMSCs were observed as spindle-shape fibroblastic morphology in FBS and HS media culture media, while cells cultured in HPL media showed spindle-like and stellate morphology. (**B**) MSCs in HPL media showed a unique swirling migration pattern throughout the study, and the cells migrated spirally and left a circular empty area in the middle of the cell colony. (**C**) Forward versus side scatter (FSC vs. SSC) plotting based on flowcytometry results showed that MSCs in xeno-free serum (HS and HPL) have a smaller cell size compared to cells in FBS media.

We examined the possibility of HPL and HS as an alternative to FBS-supplemented medium, together with other basal media, to determine a suitable culture media for a subsequent microcarrier culture. Wharton’s jelly MSCs were isolated and expanded in a total of nine media in the current study. An evaluation of P0 cell morphological characteristics under phase-contrast microscopy revealed that MSCs cultured in FBS presented a flattened and fibroblastic morphology. In contrast, MSCs cultured using HS or HPL presented a heterogeneous population of small, spindle-like, and stellate morphology (Figure 1A). MSCs cultured in HS showed similar morphology as cells cultured in HPL in the early passage, but the morphology changes to flattened fibroblastic morphology as MSCs are cultured in FBS in the later passage. A unique swirling arrangement pattern was also identified in all HPL-cultured MSCs throughout the study, and the cells were found spirally arranged and left a circular empty area in the middle (Figure 1B). A similar observation was described by Fathi Bin Hassan et al. [30] and Jonsdottir-Bush et al. [31]; however, the mechanism of this arrangement still remains unknown.

A Flow cytometry analysis of cell size and cell internal complexity (granularity) distribution profiles (FSC vs. SSC) showed an increase in cell size and granularity when cultured in FBS, and cells in xeno-free media (human serum and human platelet lysate) showed slightly smaller cell size and lower cell complexity (Figure 1C).

**Figure 2:**
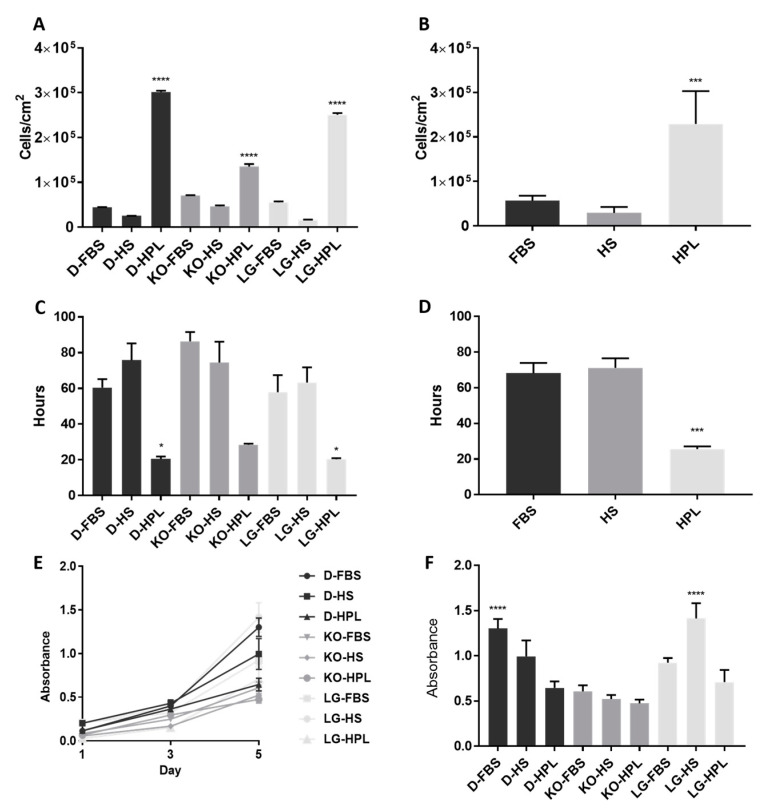
Cell proliferation and viability analysis of MSCs cultured in different culture media. The proliferation of MSCs were determined using total cell yield and population doubling time (PDT). Bar charts representing the cell yield/cm^2^ of MSCs population for (**A**) subsequent media combinations, and (**B**) average cell yield/cm^2^ for each serum supplement type. (**C**) Population doubling time for each of the MSCs culture in respective media, with (**D**) average PDT for each serum supplement type. The effects of media on the cell viability in MSCs were analysed using an MTT assay. (**E**) Cell viability cultured in different media for day 1, 3, and 5. The bar chart (**F**) representing cell viability in MSCs culture in different media on day 5 of the culture. *p* < 0.05 *, *p* < 0.001 ***, *p* < 0.0001 ****.

In general, the MSCs cultured in HPL showed a significantly higher cell yield within the basal media group (*p* < 0.05), and D-HPL had the highest cell yield overall (3.02 × 10^5^ cells/cm^2^) (*p* < 0.0001) (Figure 2A). In contrast, MSCs cultured in HS showed the lowest cell yield in each basal media group (average 2.91 × 10^4^ cells/cm^2^, D-HS = 2.5 × 10^4^ cells/cm^2^, KO-HS = 4.61 × 10^4^ cells/cm^2^, LG-HS = 1.61 × 104 cells/cm^2^), which suggests that this supplement might not be suitable for MSCs large scale expansion.

In the study of PDT, the MSCs showed a tendency to grow faster in HPL medium (PDT mean = 25.7) than in FBS medium (PDT mean = 68.2) and HS medium (PDT mean = 71.2) (Figure 2D). This shows that the addition of human platelet lysate into the commercial basal media significantly shortens the PDT of the WJMSCs (*p* < 0.0001). However, no significant difference was found in cell replicative capacity between DMEM and LG-DMEM supplemented with human platelet lysate.

The viability of MSCs cultured on the medium mentioned above was then evaluated by MTT assay. In general, the number of viable MSCs in respective media was increased from Day 1 to Day 5 (Figure 2E). However, MSCs cultured in D-FBS and LG-HS showed significantly higher viability than the others on day 5 (Figure 2F) (*p* < 0.0001).

#### 2.1.2. MSCs Cultured in LG-HPL Showed Characteristics in Accordance to the International Society for Cellular Therapy’s Guidelines

Based on the cell proliferation studies above, the MSCs cultured in LG-HPL were further validated on its characteristics due to its high cell yield and short PDT. As the effects of low mitochondrial mass remained unknown, this parameter is in consideration in this study. Even though D-HPL and KO-HPL showed similar cell proliferation capacity, low-glucose basal media is preferred for MSCs culture, as it was proven to have a greater capability to retain the plasticity of the MSCs [32,33].

**Figure 3:**
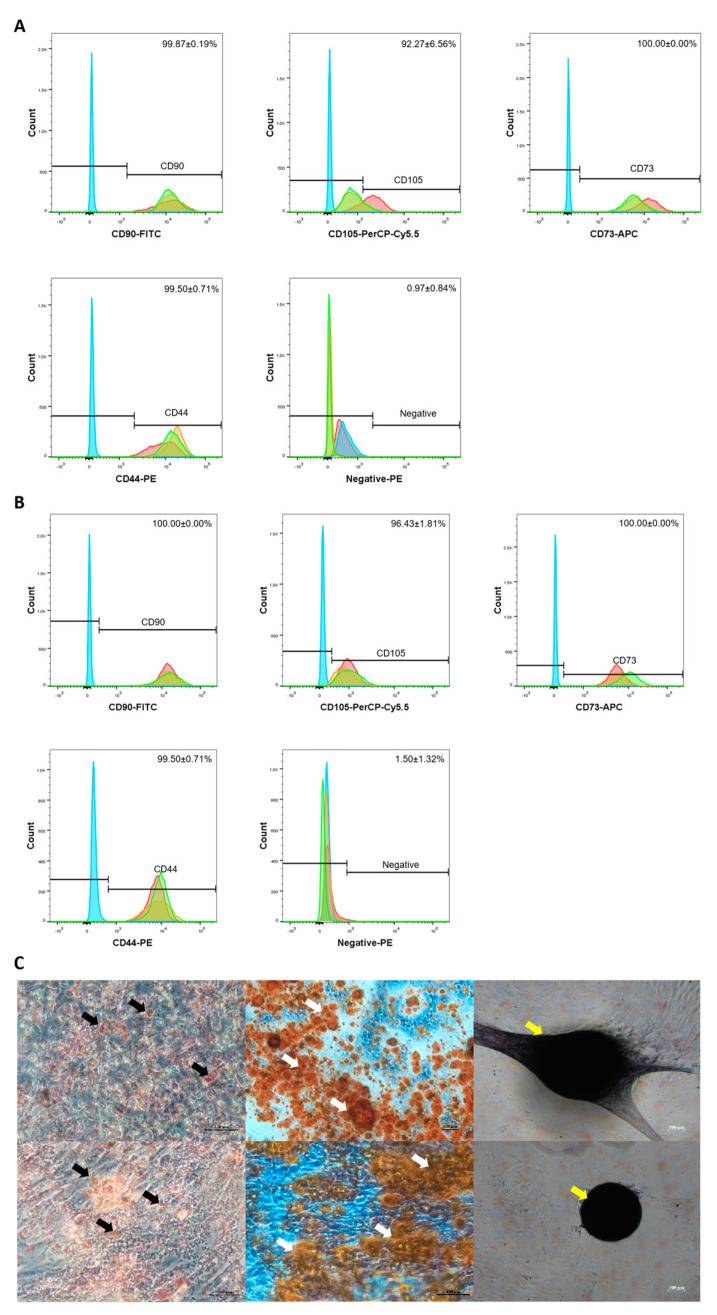
The immunophenotype and multilineage differentiation potential of MSCs monolayer culture in LG-HPL. The flow cytometry analysis shows the presence of CD90, CD44, CD105 and CD73, and the absence of CD34, CD11b, CD19, CD45 and HLA-DR (negative) in (**A**) D-FBS cultured MSCs, and (**B**) LG-HPL cultured MSCs. (**C**) The WJMSCs were cultured in adipogenic, osteogenic, and chondrogenic media for 7 (adipogenic) and 21 (osteogenic and chondrogenic) days, respectively. Lipid droplets (black arrow) observed in the cytoplasm after one week of induction through Oil Red-O staining. Osteogenic induction was assessed by Alizarin Red to detect the presence of calcium phosphate deposition (white arrow) in the extracellular matrix. Sulphated proteoglycan (yellow arrow) expression was assessed with Toluidine Blue staining. From left to right: Adipogenesis, Osteogenesis, and Chondrogenesis. Top: D-FBS cultured MSCs. Bottom: LG-HPL cultured MSCs.

The immunophenotype of MSCs were analysed by flow cytometry. As shown in Figure 3B, the high expression of MSC positive markers CD90, CD73, and CD44 were observed in the MSCs sample (100.00 ± 0.00%, 96.43 ± 1.81%, 100 ± 0.00%, and 99.50 ± 0.71% respectively). Furthermore, the negative markers (CD34, CD11b, CD19, CD45, and HLA-DR) were expressed minimally (1.50 ± 1.32%).

In vitro trilineage differentiation was performed to identify the differentiation potential of MSCs expanded in LG-HPL. The cells underwent seven days of adipogenic differentiation, and 21 days of both osteogenic and chondrogenic differentiation induction. The differentiation capacities of MSCs were examined by immunostaining with Oil Red O (lipid droplets), Alizarin Red (calcium deposition), and Toluidine blue staining (sulphated proteoglycan) (Figure 3C). Stained red lipid droplets were observed under an adipogenic induction condition. Osteogenic induction was confirmed by the presence of red calcium deposition. In chondrogenic induction, blue toluidine stain was observed in the cell pellets of the sample. The MSCs cultured in LG-HPL were thus successfully characterised according to the minimal criteria for defining multipotent mesenchymal stem cells, as mentioned by the ISCT [34]. The MSCs displayed a spindle shape, expressed all the MSC markers, and were able to differentiate into adipocytes, osteocytes, and chondrocytes. The high expansion ability observed in MSCs cultured in LG-HPL was similar to that in other studies [30,35,36,37,38]. This further demonstrates that this xeno-free media formulation is suitable for the expansion of MSCs.

### 2.2. WJMSCs Microcarrier Culture in Xeno-Free Culture Condition

#### 2.2.1. MSCs Microcarrier Culture in LG-HPL Media Showing Stagnated Proliferation

**Figure 4:**
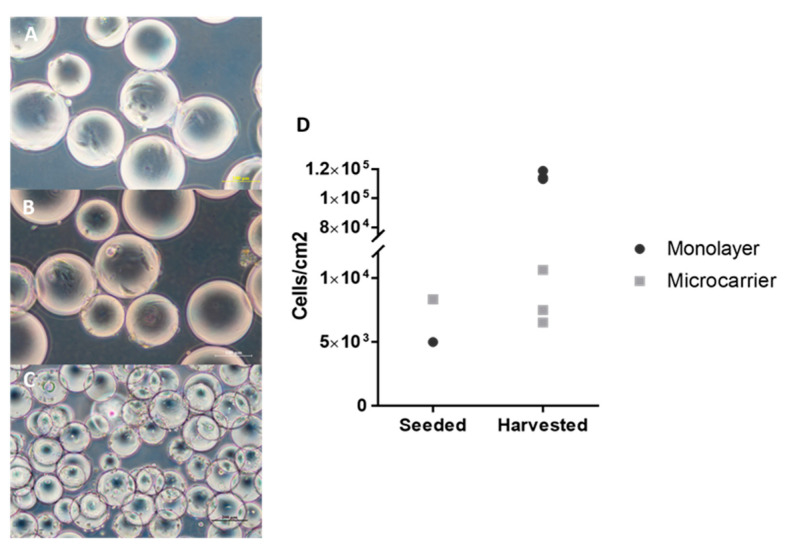
WJMSCs microcarrier culture in spinner flask. (**A**–**C**) Morphological changes of MSCs microcarrier culture on day 1, 3, and 5 (from top to bottom). (**D**) The proliferation of MSCs in monolayer culture and microcarrier culture was compared by total cell yield/cm^2^.

In order to identify the cell behaviour in three-dimensional culture conditions, MSC cultures were performed in spinner flasks containing Cytodex-1 and agitated at the NJS, where NJS is the impeller speed at which the microcarriers are ‘just fully suspended’ by visual observation [39], and 30 rpm was chosen for this experimental setup. The expansion of MSCs on microcarriers was achieved over five days, where the MSC concentration reached a maximal value of 1.06 × 104 cells/cm^2^ (Figure 4D). The microscopic observations throughout the culture period were as follows. With the optimised inoculation strategy, uniform cell attachment was achieved in the Day 1 culture. Further examination of the culture on Days 2–4, however, revealed that the MSCs attached to the microcarrier were slowly detaching (rounded) (Figure 4A,B), and no cell proliferation was observed; cell detachment was observed from Day 5 (Figure 4C).

**Figure 5:**
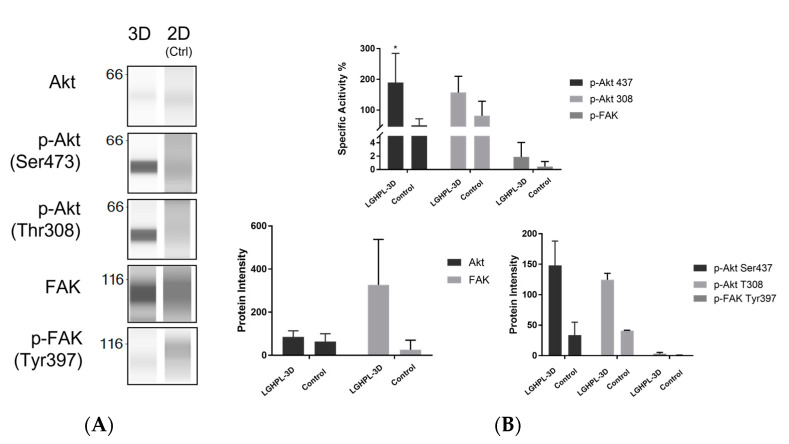
The expression levels of Akt, *p*-Akt (Ser473), p-Akt (Thr308), FAK, and p-FAK (Tyr397) in WJMSCs monolayer/ microcarrier culture as determined by a Jess™ Simple Western automated nano-immunoassay system. (**A**) Representative image of Akt, p-Akt (Ser473), p-Akt (Thr308), FAK, and p-FAK (Tyr397) protein levels from Jess simple western blotting. (**B**) Specific activity of p-Akt (Ser473), p-Akt (Thr308), and p-FAK (Tyr397) to determine the phosphorylation of Akt and FAK in WJMSCs monolayer and microcarrier culture. *p* < 0.05 *.

Figure 5A presents immunoblot and densitometry analyses of MSCs cultured on microcarriers. Total Akt expression was found to be highly expressed in MSCs cultured on microcarriers compared to MSCs in the monolayer. A densitometry analysis of Akt showed significant differences (*p* < 0.05) between them. A densitometry analysis of Akt activation via phosphorylation at Ser473 and Thr308 showed a significant difference compared to the control. The phosphorylation of FAK for both control and microcarrier culture, however, is relatively low (Figure 5B).

#### 2.2.2. MSC Microcarrier Cultures Retained Their Characteristic as MSCs after Being Cultured on Microcarriers

**Figure 6:**
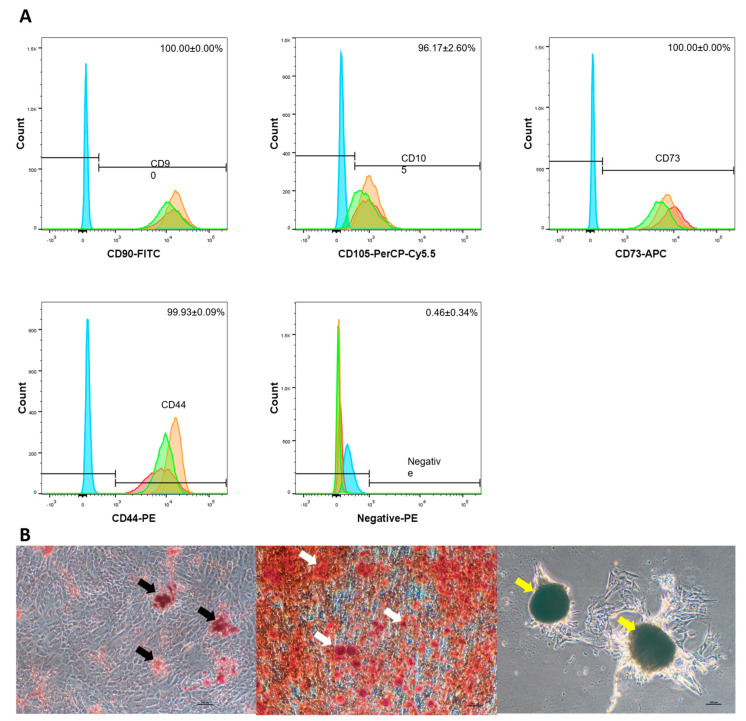
The immunophenotype and multilineage differentiation potential of MSCs microcarrier culture in LG-HPL. The flow cytometry analysis shows the presence of CD90, CD44, CD105 and CD73, and the absence of CD34, CD11b, CD19, CD45 and HLA-DR (Negative) in (**A**) LG-HPLMSCs microcarrier culture. (**B**) The WJMSCs were cultured in adipogenic, osteogenic, and chondrogenic media for 7 (adipogenic) and 21 (osteogenic and chondrogenic) days, respectively. Lipid droplets (black arrow) observed in cytoplasm after one week of induction through Oil Red-O staining. Osteogenic induction was assessed by Alizarin Red to detect the presence of calcium phosphate deposition (white arrow) in the extracellular matrix. Sulphated proteoglycan (yellow arrow) expression was assessed with Toluidine Blue staining. From left to right: Adipogenesis, Osteogenesis, and Chondrogenesis.

Even though the proliferation of MSCs remained lower in the microcarrier culture, the harvested viable cells retained their characteristics. The MSCs harvested revealed the high expression of MSC positive markers CD90, CD73, and CD44, and the minimal expression of negative markers (CD34, CD11b, CD19, CD45, and HLA-DR) (Figure 6A). No significant difference was observed between the expression of MSC surface markers in a monolayer culture and a microcarrier culture. The differentiation potential of these harvested cells towards adipocyte, osteocytes, and chondrocytes was then explored: the MSCs cultured on microcarriers were able to differentiate, as expected, as demonstrated by specific staining with Oil red O (adipogenesis), Alizarin red (Osteogenesis), and Toluidine blue (chondrogenesis) (Figure 6B). Again, no significant differences were observed between the monolayer culture and the microcarrier culture.

#### 2.2.3. MSCs Microcarrier Culture Maintaining High Inhibitory Activity on TNF-α and IL-1 Secretion by PBMCs

**Figure 7:**
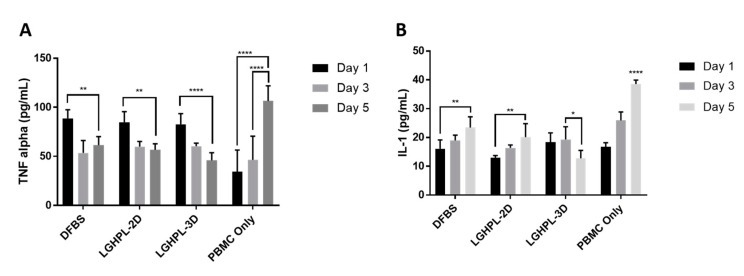
Immunomodulatory activity of WJMSCs on the secretion of TNF-α. The immunomodulatory activity of WJMSCs was evaluated by co-culturing with activated PBMCs. Bar chat representing the concentration of (**A**)TNF-α and (**B**) IL-1 secreted by PBMCs on day 1, 3, and 5. *p* < 0.05 *, *p* < 0.01 **, *p* < 0.0001 ****.

In addition to the characteristics mentioned above, MSCs are widely known for their strong immunomodulatory properties. Many inflammatory-related clinical trials involving MSCs have thus been conducted. The immunomodulatory effect of MSCs was therefore examined in vitro. An inflammatory environment was simulated by activating PBMCs in vitro with PHA. MSCs exposed to activated PBMCs resulted in the significant inhibition of TNF-α in monolayer culture (*p* < 0.05), and the MSC microcarrier culture showed greater TNF-α inhibition compared to the monolayer culture (*p* < 0.0001). (Figure 7A). The concentration of TNF-α significantly decreased on Day 3, but the suppression remained insignificant on Day 5 of the culture. In the case of IL-1, significant suppression was observed by MSC microcarrier culture on Day 5 (*p* < 0.05). In contrast, while no suppression was observed, the secretion of IL-1 increased significantly in the MSC monolayer culture (*p* < 0.05) (Figure 7B).

## 3. Discussion

In this study, we evaluated the potential of a xeno-free serum supplement in replacing FBS for future clinical application. We also identified the growth of MSCs in xeno-free culture media under the microcarrier culture system.

Based on the cell yield and PDT of the MSCs, HPL was found to be superior to FBS and HS in promoting cell proliferation. The total cell yield using HPL was significantly higher within its basal media group, and the average PDT of MSCs cultured in HPL is significantly lower than when using the other two supplements (Figure 2D). The trend observed in the aforementioned results was most likely due to the HPL composition that essentially differs from FBS, which is significantly higher in amounts of cell proliferation-support growth factors such as IGF-1 (insulin-like growth factor 1) and PDGF (platelet-derived growth factor) [40]. The cell yield differences of up to 7.8 times between HPL cultured MSCs and the other two supplements was believed to be due to the smaller size of the HPL cultured MSCs, therefore more cells can be occupied in the same surface area of the culture flask. This could actually be one of the cell expansion strategies; as the size of the MSCs reduced, higher cell yield can be expected due to the higher cells:cm^2^ ratio in the cell culture surface, while the cell viability of the MSCs cultured in HPL-supplemented media is lower than those in FBS and HS. The cause of low viability could most probably be due to the reduction of mitochondrial mass, as a study reported that the HPL supplement reduced the mitochondrial mass of the monolayer cultured amniotic mesenchymal stem cells [41]. Since the MTT reduction was dependent on the mitochondrial dehydrogenase activity of the cells instead of the direct reflection of cell numbers, a lowered mitochondrial mass could lead to reduced dehydrogenase activity in the assay, reflecting the low viability on HPL cultured MSCs. For an effective clinical application of MSCs, the effects of HPL on MSCs mitochondrial-related regulatory pathways and biogenesis have to be further explored, as there are limited studies that report on this observation.

Even though MSCs cultured in HPL medium in monolayer culture showed excellent cell expansion, the MSCs microcarrier culture suggested a contradictory result. There was no apparent nutrient limitation in the culture, as 50% of the media was changed every two days, and the results observed in this study contradict others. Hewitt et al. observed maximal cell density achieved after 8–10 days with an up to 20-fold expansion of cells per microcarrier [42]. Yan et al. also demonstrated greater MSC expansion to 500-fold, with a final yield of 1.05 ± 0.11 × 10^9^ cells achieved [43]. These results demonstrate that the MSCs microcarrier culture in this study have a relatively low proliferation compared to other studies. In order to elucidate the underlying mechanisms mediating this situation, we assessed the changes in the expression of a few signalling molecules related to cell adhesion and proliferation. The PI3K/Akt signalling pathway plays a critical role in the survival, proliferation, migration, angiogenesis, cytokine production, and differentiation of MSCs. Studies revealed that the overexpression of Akt improved the survival of MSCs, especially after in vivo transplantation [44,45,46,47]. The transplanted PI3K/Akt pathway-activated MSCs were found to have greater survival in the rat model of myocardial infarction [48]. A similar study also reported the activation of the PI3K/Akt pathway by LPS-protected MSCs from oxidant stress-induced apoptosis [49]. The activation of the PI3K/Akt pathway by multiple growth factors also demonstrated an increment in MSC proliferation. PGE2, for example, stimulated MSCs proliferation via βcatenin-mediated c-Myc and VEGF expression by Akt-specific activity [50]. The high specific activities of *p*-Akt sub-units in MSC microcarrier culture in this experiment suggested that the cells remained highly viable, while the low proliferation could be due to other factors, such as failure in cell adhesion.

The adhesion of MSCs on microcarriers has a direct effect on the expansion efficiency. While FAK is a widely expressed cytoplasmic protein tyrosine kinase that involves cell adhesion and migration [51,52,53,54], the low specific activity of p-FAK observed in this study could be one of the factors leading to low cell proliferation on microcarrier culture. Studies revealed that the overexpression of tTG increased the phosphorylation of focal adhesion-related kinases, including FAK in rat MSCs, which resulted in a successful implantation that restored cardiac function in a rat model [55]. It also showed that the generation of ROS interrupted the activation of focal adhesion-related kinases, resulting in cell death in transplanted MSCs [56,57,58]; these observations showed the association between FAK activities and MSCs survivability.

Anoikis is a subset of apoptosis in cells upon the loss of attachment to the extracellular matrix (ECM) and neighbouring cells [59,60,61,62]. The relationship between cell adhesion and apoptosis was first elucidated and defined by Steven M. Frisch and Hunter Francis in 1994 [63]. As the FAK signalling pathway is closely related to cell adhesion, it was found that phosphorylation of the FAK dependent pathway greatly suppresses anoikis induction in adherent cells [64,65,66,67]. Examining the low cell adhesion potentially caused by inactive FAK activities in this study suggests that there is a high possibility that anoikis was induced in the MSCs cultured on microcarriers. Several modifications can be considered to improve cell adhesion/proliferation on microcarriers. As mentioned above, the activation of FAK dependent pathways could improve cell adhesion, and thus the FAK activator/restriction of FAK inhibitor in the culture system could be involved. Mo et al. discovered that the overexpression of the IQGAP1 gene leads to anchorage-independent growth and promotes phosphorylation of the Src/FAK pathway [68]. A study also showed that the exposure of low-intensity pulsed ultrasound towards human adipose-derived MSCs improved cell adhesion and proliferation, which suggested a new approach to MSC pre-treatment prior to microcarrier culture [69]. The selection of microcarriers also affects the adhesion of MSCs in the culture system, as the fabrication material differs [70]. This might be because the surface charges present on Cytodex 1 in this experiment could only support cell adhesion at the beginning of the inoculation period, while during the culture phase, the stirring of culture vessels created potential shearing forces applied to the cells, and also collision forces applied randomly due to the agitation of microcarriers; these forces could contribute to the cell detachment and lead to anoikis. In this case, microcarriers with surface modification (greater surface charges, chemical/ biocompatible material coating) could be a solution to strengthening the cell adhesion on microcarriers.

Our study also demonstrated the potential of MSCs microcarrier culture in modulating inflammatory events. TNF-α is the most widely studied cytokine within the tumour necrosis factor superfamily [71,72]. It is a potent paracrine and endocrine mediator of inflammatory and immune functions, primarily released during acute phase reactions or innate immune responses. The TNF-α associated signalling pathway involved a series of phosphorylation and ubiquitination events, and breaking the balance of these events resulted in the activating of a chain reaction of TNF-α upregulation coupled with an aggressive inflammatory response due to releases of a large amount of pro-inflammatory cytokines. Similar to TNF-α, IL-1 is a potent inflammatory cytokine that plays a crucial role in innate immunity. The secretion of IL-1 by mast cells or macrophages activates a series of pro-inflammatory cytokines, contributing to adverse inflammatory reactions. Excess IL-1 activities were found in numerous situations, including autoinflammatory events [73,74,75,76,77], metabolic syndromes [78,79,80,81], acute/chronic inflammation [82,83,84,85] and malignancy [83,86,87]. In the current COVID-19 virus pandemic, the viruses were exerted not only through direct cytotoxic mechanisms but also by triggering cytokine storms that cause severe Grade 4 life threatening cytokines release syndrome [88]. Studies have demonstrated that the correlation of TNF-α and IL-1 with critical COVID-19 cases [82,84,89,90], and the immunomodulation of TNF-α and IL-1 by MSCs observed in this study could be a potential treatment for reducing COVID-19 mortality.

## 4. Materials and Methods

### 4.1. WJMSC Isolations and Culture

The material used in the study comprised the umbilical cord (*n* = 3) obtained from the Universiti Kebangsaan Malaysia Medical Centre with informed consent from donors who delivered full term by elective Caesarean section. Healthy donors (below 40 years-of-age) without any complications during the pregnancy were chosen as the target for sample collection. The umbilical cord was washed with Dulbecco’s phosphate-buffered saline (DPBS) (Gibco, San Diego, CA, USA) and stripped of the umbilical arteries and veins. Wharton’s jelly was minced into a 2 mm^2^ size and digested with 0.6% collagenase type II (Worthington, Lakewood, NJ, USA) at 37 °C under gentle agitation. The digested tissues were centrifuged and the pellet was resuspended in respective complete culture medium (Table 1). The cells were cultured in a humidified incubator with 5% CO_2_ at 37 °C with medium change every 3 days until the cells were expanded and harvested using 1X TrypLE Select (Gibco, San Diego, CA, USA).

### 4.2. Calculation of Population Doubling Time (PDT)

The calculation of population doubling time (PDT), was carried out as described by Miyazawa et al. (2010) [91]. Briefly, cells were seeded in triplicate at a concentration of 5000 cells/cm^2^ and cultured as described. PDT, measured in hours, was calculated according to the following equation: PDT = tlog2/(logN2 − logN1), where t denotes time in culture; N2 denotes cell number at the end of the passage; and N1 denotes the cell number seeded at the beginning of the passage.

### 4.3. Analysis of Cell Viability

The isolated cell’s viability has been analysed by 3-(4,5-dimethylthiazoiyl-2)-2,5-diphenyltetrazolium bromide (MTT) coloration (Invitrogen, Waltham, MA, USA). The cells were seeded in a density of 5000 cells/cm^2^, and MTT assays were done on day 1, 3, and 5 to determine the viability of the MSC cultured in the aforementioned medium. The assay was performed in accordance with the manufacturer’s instructions.

### 4.4. Microcarrier Preparation

Cytodex-1 (Sigma-Aldrich, St. Louis, MO, USA) was prepared in accordance with the manufacturer’s instructions. In brief, the microcarriers were hydrated in Ca^2+^ and Mg^2+^ free DPBS for at least 3 h at room temperature. The supernatant was discarded and the microcarriers were washed with fresh Ca^2+^ and Mg^2+^ free DPBS for 3 min. The supernatant was then discarded again and replaced with fresh Ca^2+^ and Mg^2+^ free DPBS prior to autoclaving. A total of 120 mg of Cytodex 1 was used for each spinner flask culture, which has a total surface of 360 cm^2^ per flask (according to the manufacturer’s information). The microcarriers were then pre-conditioned in 30 mL of complete culture media in a humidified incubator with 5% CO_2_ at 37 °C prior inoculations.

### 4.5. WJMSCs Microcarrier Culture

Passage 4 WJMSCs were harvested as mentioned above, and a total of 3 × 10^7^ WJMSCs in 20 mL of complete culture media were seeded into each spinner flask. The microcarrier culture was agitated in 125 mL Corning^®^ Disposable Spinner Flasks (Corning, US) with an external magnetic stirring system. The inoculation phase comprised a 5 min stirring at 30 rpm, followed by 45 min static culture. After 12 h of inoculation, the culture medium was topped up to 100 mL, and the agitation was set to 30 rpm continuously. The cells were cultured in a humidified incubator with 5% CO_2_ at 37 °C, with 50% medium change every 2 days until the cells were harvested. On day 5, the cell harvest was carried out as described by Nienow et al. (2014) [92] with slight modifications. Briefly, the spinner flask was removed from the magnetic stirring system, and the cells-microcarrier aggregates were allowed to settle for 5 min. The cells were then rinsed with DPBS under agitation (30 rpm) and incubated with 35 mL 1X TrypLE Select (Gibco, San Diego, CA, USA) under agitation (150 rpm) for 7 min at 37 °C.

### 4.6. WJMSCs Immunophenotypic Analysis and Differentiation Analysis

The immunophenotype of WJMSCs was determined by flow cytometry using a Human MSC Analysis Kit (BD, San Diego, CA, USA). In brief, the cells were incubated with the following antibodies in accordance with the manufacturer’s protocol: FITC CD90, PerCP-Cy™5.5 CD105, APC CD73, PE CD 44, PE CD45, PE CD34, PE CD11b, PE CD19, and PE HLA-DR. The cell analysis was performed using FACSVerse™ to determine the expression of cell surface markers.

Lineage differentiation of WJMSCs was determined according to Sulaiman et al. [93] with slight modifications; the cells were cultured in differentiation induction media for multilineage differentiation. Osteogenic differentiation was induced for 21 days in StemPro^®^ Osteogenesis Differentiation media (Gibco, San Diego, CA, USA). The induced cells then had a medium change every 3 days. Osteogenic differentiation activity was accessed with Alizarin Red staining, which positively stained for calcium deposition in the extracellular matrix of the cells. In adipogenic differentiation, WJMSCs were cultured in StemPro^®^ Adipogenesis Differentiation media (Gibco, San Diego, CA, USA). The induced cells then had the medium changed every 3 days for 7 days. The samples were stained with Oil Red O to identify lipid deposition within the cytoplasm. In chondrogenic differentiation, WJMSCs were cultured in StemPro^®^ Chondrogenesis Differentiation media (Gibco, San Diego, CA, USA). The induced cells then had the medium changed every 3 days. The resulting culture was fixed and stained with Toluidine Blue to identify the presence of proteoglycans using a bright field microscope.

### 4.7. Immunosuppression Assay

To prepare activated peripheral blood mononuclear cells (PBMCs), anticoagulant-treated blood was diluted with DPBS and added into SepMate™ PBMC Isolation Tubes (STEMCELL Technologies, Canada) with Ficoll Paque PLUS (Cytiva, Marlborough, MA, USA) added. After centrifugation, the PBMC layer was collected and washed by adding DPBS and centrifuged at 100× *g* for 10 min at room temperature. The PMBC pellet was resuspended with RPMI 1640 supplemented with 1 % GlutaMax, 1% Anti-anti, 10% FBS, and 20 µg/mL phytohemagglutinin (PHA) for activation.

The activated PBMCs were then co-cultured with WJMSCs in a 1:10 ratio (MSCs: PBMCs). The concentration of inflammatory cytokines, Tumour Necrosis Factor alpha (TNF-α), and Interleukin 1 (IL-1) was then quantified via ELISA after day 1, 3, and 5 of co-culture.

### 4.8. Western Blot Analysis

The Jess™ Simple Western automated nano-immunoassay system (ProteinSimple, San Jose, CA, USA, a Bio-Techne Brand), a capillary-based size separation of proteins, was used. Cell lysate samples were processed according to the manufacturer’s standard method for the 12–230-kDa Jess Separation Module (SM W002) and Jess Fluorescence separation module (SM-FL001-006). Briefly, a mixture of cell lysate samples, fluorescent molecular weight markers, and 400 mM dithiothreitol (Protein Simple) was prepared at a final concentration of 1.2 μg/μL and then denatured for 5 min at 95 °C. Primary antibodies, AKT #MAB2955 (R&D Systems, Minneapolis, MN, USA), phospho-AKT S473 34060T (Cell Signalling Technology, Danvers, MA, USA), phospho-AKT1 #MAB7419 (R&D Systems, Minneapolis, MN, USA) FAK #MAB4467 (R&D Systems, Minneapolis, MN, USA), and phospho-FAK (Cell Signalling Technology, Danvers, MA, USA) were diluted in milk-free antibody diluent 2 buffer (Protein Simple, #042-203), while the secondary antibodies (Protein Simple, #042-206 and #043-206) were ready to use. In total, 10 μL of primary and secondary antibodies were loaded for each sample. Peroxide/luminol-S (ProteinSimple) was used for chemiluminescent revelation. To remove bubbles, the plate was spun-down for 5 min at 1000× *g*. Subsequently, the capillaries and plate were loaded into the Jess machine. Protein separation, blocking, antibody incubation and signal detection were undertaken automatically by a Jess system. Data were analysed using The Compass Simple Western software (version 6.1.0, ProteinSimple) to calculate the heights (chemiluminescence intensity), area, and signal/noise ratio, as well as to capture the digital image of the capillary chemiluminescence.

### 4.9. Statistical Analysis

Data were represented as mean ± SD of at least three biological replicates (*n* = 3). Data were analyzed using Prism Version 7.0 software. A one-way ANOVA and Tukey’s post hoc test were conducted for pairwise comparisons. Results were considered statistically significant at *p* value less than 0.05 (*p* < 0.05).

## 5. Conclusions

The challenges associated with conventional culture methods and clinical applications mean that the venture into xeno-free three-dimensional culture systems has garnered increasing interest due to the potential mass production of safe, zoonotic transmission-free MSCs. In conclusion, LG-HPL was identified as a good xeno-free media for WJMSC culture, without compromising the characteristics of the cells, as mentioned by ISCT. The proliferation of these MSCs has been identified, revealing the potential for the up-scale production of MSCs in xeno-free culture conditions. The results obtained from the studies above were translated into microcarrier culture systems; however, stagnated MSC proliferation was observed, and cell detachment occurred during the five day culture period. The activities of Akt and FAK were identified by western blot to elucidate the cause of low cell proliferation and cell detachment of MSCs on the microcarrier culture. The MSC microcarrier culture was found to have inactive FAK activity, suggesting that the cells underwent anoikis, which led to cell detachment, as mentioned above. Nonetheless, the characteristics of MSCs in microcarrier culture remained, and an immunomodulatory study revealed that the MSCs cultured in microcarrier culture have high suppression on TNF-α and IL-1, showing the potential of xeno-free three-dimensional system cultured MSCs as a treatment in inflammatory events in the current COVID-19 pandemic. As the three-dimensional microcarrier culture appeared to be a great potential culture system for MSC expansion, there is an increasing need to understand the proliferation mechanism and signalling pathway of MSCs in a three-dimensional culture system. The importance of the culture surface has led to increasing efforts to develop various systems to fulfil this need. While the microcarrier culture was one of the innovations, the fundamental concept of providing an adherent surface for MSCs proliferation remained unchanged. The modification on a microcarrier surface and precise agitation strategies should therefore be extensively studied to improve the expansion capacity of MSCs. A better understanding of each of the factors mentioned will improve the expansion process of MSCs and contribute a standardised practise for MSC three-dimensional culture.

## Figures and Tables

**Table 1:** List of basal media with serum supplements for WJMSCs monolayer culture.

Basal Media	Serum	Supplements
Dulbecco’s Modified Eagle’s Medium (DMEM) (Gibco, San Diego, CA, USA)	Foetal Bovine Serum (Sigma-Aldrich, St. Louis, MO, USA)	1% GlutaMax (Gibco, San Diego, CA, USA) + 1% Antibiotic & Antimycotic (Corning, NY, USA)
	Human Platelet Lysate (Merck, Rahway, NJ, USA)	
	Human Serum (Sigma-Aldrich, St. Louis, MO, USA)	
DMEM-Knockout (KO) (Gibco, San Diego, CA, USA)	Foetal Bovine Serum (Sigma-Aldrich, St. Louis, MO, USA)	
	Human Platelet Lysate (Merck, Rahway, NJ, USA)	
	Human Serum (Sigma-Aldrich, St. Louis, MO, USA)	
DMEM Low Glucose (LG) (Sigma-Aldrich, St. Louis, MO, USA)	Foetal Bovine Serum (Sigma-Aldrich, St. Louis, MO, USA	
	Human Platelet Lysate (Merck, Rahway, NJ, USA)	
	Human Serum (Sigma-Aldrich, St. Louis, MO, USA)	

## Data Availability

Not applicable.
